# Acute fatal delayed subdural hemorrhage diagnosed with head computed tomography scan 19 days after hospital admission: A case report

**DOI:** 10.1016/j.radcr.2026.06.027

**Published:** 2026-07-02

**Authors:** Khalid Ahmed, Gustav Strandvik, Ayman El-Menyar, Tariq Siddique, Ruben Peralta, Mohammad Asim, Hassan Al-Thani

**Affiliations:** a Trauma Surgery, Department of Surgery, Hamad General Hospital, Doha, Qatar; b Faculty of Medicine, Qatar University, Doha, Qatar; c Clinical Medicine, Weill Cornell Medicine, Doha, Qatar; d Department of Surgery, Universidad Nacional Pedro Henriquez Urena, Santo Domingo, Dominican Republic

**Keywords:** Traumatic, Hematoma, Subdural, Intracranial hemorrhage, Decompressive craniectomy

## Abstract

Delayed acute traumatic subdural hematoma (SDH) refers to an acute SDH that develops after an initially normal head computed tomography (CT) scan in a trauma patient. Although uncommon, it occurs more often in elderly patients and in those receiving anticoagulation therapy. However, current traumatic brain injury monitoring guidelines may not always account for delayed intracranial bleeding in younger patients without typical risk factors. We report a 42-year-old male involved in a motor vehicle collision. The initial CT images demonstrated chest injuries and a normal head CT. The patient was not receiving anticoagulant or antiplatelet therapy. While recovering from his traumatic injuries, on hospital day nineteen, he acutely deteriorated and was found to have a large acute SDH. Despite emergent decompressive craniectomy and hematoma evacuation, the patient died. This case highlights that a normal head CT in trauma patients on admission does not exclude the possibility of a subsequent catastrophic intracranial hemorrhage. Such unpredictable consequences may occur days or weeks after an apparent clinical recovery.

## Background

Mild traumatic brain injury (TBI) usually requires a period of observation, but less than 1% requires neurosurgical intervention because of delayed intracranial hemorrhage [1,2]. Delayed acute subdural hematoma (SDH) is defined as an acute SDH that is not apparent on the initial CT but becomes evident on a follow-up CT [3]. It is usually more common in elderly patients and/or on anticoagulation or antiplatelet therapy [4]. While in about 70 percent of patients [1]. Neurologic deterioration will be evident within the first 24 hours, but delayed deterioration in the conscious level after improvement from the initial nonhead injuries is rare. We present a young male patient with traumatic chest injuries and a normal head CT scan on admission. He developed delayed acute SDH 19 days after hospitalization.

## Case description

This is a 42-year-old male who was involved in a motor vehicle collision. At the scene, he was found in respiratory distress with a deep, irregular respiratory rate of twenty breaths per minute and a low Glasgow coma scale (GCS) score, likely due to alcohol intoxication. Consequently, he was intubated at the scene and transported to the trauma room. Upon examination in the trauma room, he had a GCS score of 2T/15, which indicated severe impairment, and bilateral 3 mm reactive pupils, suggesting initially intact brainstem function despite the severe condition. Under the effects of a muscle relaxant and sedation, the chest examination revealed decreased air entry on the left side, while the FAST exam was negative. A CT scan showed multiple left-sided rib fractures with underlying pulmonary contusions, mild hemothorax, and pneumothorax, with no abnormalities in the head CT scan (Fig. 1), abdomen, and pelvis. The ethanol level was 65.1 mmol/l, reflecting significant intoxication at the time of the incident. He was subsequently admitted to the trauma ICU. A follow-up CT head the next day (Fig. 2) revealed a small extra-axial hematoma, a few scattered small hemorrhagic contusions, and minimal subarachnoid hemorrhage. Due to persistent low GCS, an MRI on day 3 showed a diffuse axonal injury grade 3. The patient was started on DVT prophylaxis and maintained on levetiracetam 500mg, initiated at admission. A subsequent CT scan the next day showed no significant changes. Due to persistently low GCS, he underwent percutaneous dilatation tracheostomy on the seventh day of his admission.

**Fig. 1:**
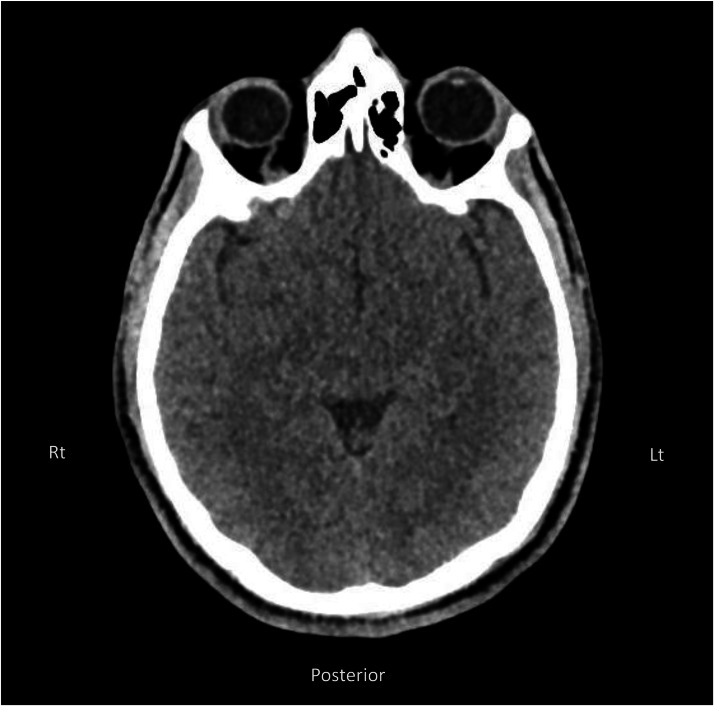
Day 1 postadmission plain head CT scan shows no cerebral pathology identified (Slice thickness of 1.50 mm).

**Fig. 2:**
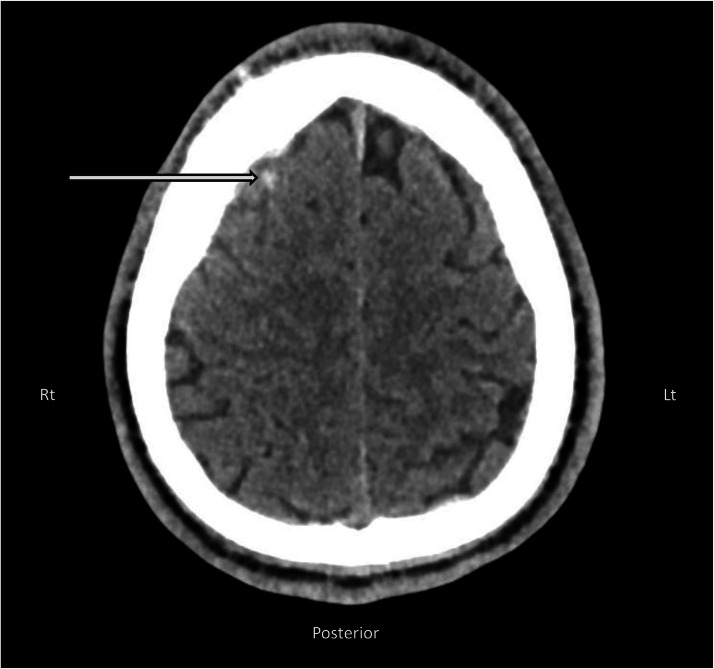
Day 2 postadmission plain head CT scan shows right small frontal contusion (Slice thickness of 1.50 mm).

Over the following week, his GCS improved to E3M6Vt. He was tolerating a tracheal mask. Over the next 10 days, his conscious level improved further, with a GCS of E4M6 Vt. On the 19th of his admission, in the evening, he suddenly became unresponsive with fixed dilated pupils with no corneal reflex but preserved cough reflex. There was no witnessed or suspected fall, and no abnormal behavior or agitation before the incident.

The patient underwent emergency CT head showing newly developed right-sided cerebral convexity SDH with diffuse edema, with midline shift of 7.5 mm toward the left side and crowding at the foramen magnum with tonsillar herniation (Fig. 3). He underwent emergency right decompressive craniectomy and evacuation of acute SDH (Fig. 4). The presumed source of the hematoma was the cortical vein, which was identified and coagulated. After returning from the operating room, on examination, his pupils were fixed and dilated, with no corneal or cough reflex. He underwent a follow-up CT scan of the head that showed findings suggestive of multiple foci of ischemic changes, diffuse edema, and brainstem hemorrhage. The next day, the patient was polyuric with a diagnosis of diabetes insipidus. As he lost the brain stem reflexes, the brain stem testing confirmed brain death on the third day of the event.

**Fig. 3:**
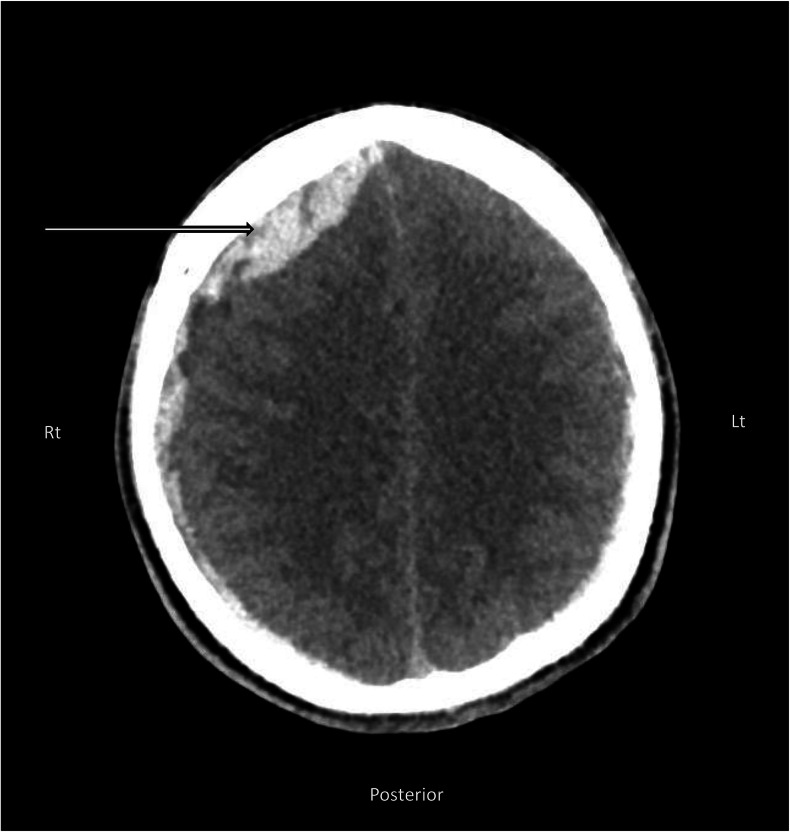
Day 19 postadmission plain head CT scan shows acute right extra axial hemorrhage (arrow).

**Fig. 4:**
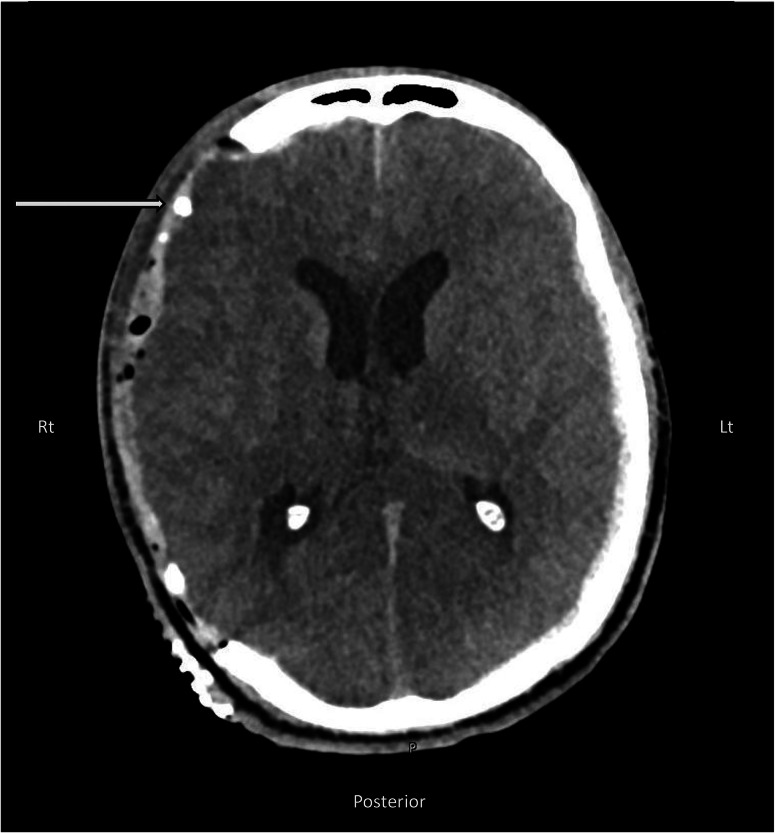
Day 20 CT head (postoperative) shows post right-sided decompressive craniectomy (arrow).

## Discussion

In the setting of trauma, a GCS score of 13-15 points is defined as mild TBI. It will require observation for at least 24 hours; if there is any suspicion of deterioration, but less than 1% require neurosurgical intervention [1,5]. According to current Brain Trauma Foundation guidelines, observation and monitoring recommendations typically focus on a shorter 24-hour window postinjury, with further neuroimaging guided by early clinical changes. However, our case demonstrates that catastrophic delayed intracranial hemorrhage can occur far beyond this standard observation period, with deterioration presenting as late as nineteen days after the initial trauma and a negative CT scan. This stark contrast underscores the potential need to reconsider observation protocols and increase clinician vigilance even after the traditional monitoring window.

However, in rare cases, delayed intracranial hemorrhage may occur [2]. Proposed mechanisms underlying delayed intracranial hemorrhage, including vessel wall weakening, increased venous pressure, and congestion, have been reported [6]. Recent studies have suggested complex, interrelated processes underlying membrane formation, angiogenesis, fibrinolysis, and inflammation during the development of delayed-onset SDH [5].

Although the negative predictive value of a head CT read as preliminary negative for neurosurgical intervention is around 99.7 percent [7], documented case reports in the literature of acute subdural bleeds occurring more than 2 weeks after the head CT that resulted in mortality are extremely rare (Table 1) [5,6,[8], [9], [10]]. To our knowledge, few published cases report a delay exceeding 14 days between trauma and the development of fatal acute subdural hematoma, underscoring the rarity and clinical importance of such long-term delayed presentations.

**Table 1:** Summary of recent literature on delayed subdural hemorrhage cases.

Study	Age/sex	Comorbidities	Mechanism of trauma	Arrival presentation	GCS on arrival	Follow-up CT findings	Days of SDH	Possible cause	Interventions	Outcome
Nagpal et al. [9]	72 y male	Unremarkable	Minor fall	Giddiness, headache, balance issues	Not stated	Bilateral SASDH, cerebral edema	∼30 d	Minor trauma/insidious onset	Bilateral mini craniotomy	Satisfactory
Khulsai et al. [10]	23 y male	Unremarkable	Traffic related (3 years prior)	Headache, memory loss, slurred speech, urinary incontinence	Not stated	Large left fronto-parietal mass (CSDH)	∼1,095 d (3 y)	Late sequel of old traumatic head injury	Surgical evacuation and excision	Successful resolution
Chia et al. [5]	84 y male	None (no anticoagulants)	Minor head trauma	Dizziness, vomiting (initial); Unsteady gait (2 months later)	Normal initially	Large right CSDH	∼60 d	Delayed evolution from minor trauma	Emergency burr-hole evacuation	Discharged without deficit
Chen et al. [8]	48 y female/65 y male	Healthy; Chest trauma	Traffic; Tumble	Alert/Oriented	15	Thin SDH/SAH	10 d; 9 d	Rapid progress of minor SDH	Decompressive craniotomy	Death (both)
Matsuda et al. [6]	18 y male	None	Football injury (occipital)	Sudden splitting headache	15	Day 6: Left ASDH. Day 14: Subacute SDH	6 d	Straining (Valsalva)/delayed liquefaction	Hematoma irrigation with trephination	Complete recovery
Present case	42 y male	Alcohol intoxication	motor vehicle collision	Respiratory distress	Intubated	Right SDH	19 d	Not known	decompressive craniectomy and evacuation	Died

ASDH, acute SDH; CSDH, chronic SDH; ICH, intracerebral hemorrhage; SASDH, sub-acute subdural hematoma; SDH, subdural hematoma.

Our patient showed signs of clinical improvement during the hospital stay, but suddenly, on the day 19 postadmission, his GCS dropped with the development of acute subdural hematoma. Despite undergoing emergency surgery, his life could not be saved. Of note, blood tests on admission revealed alcohol intoxication, a possible risk factor for further investigation. Prior report revealed the potential alcohol-induced increase in bleeding risk [11]. Alcohol consumption can impair primary hemostasis by reducing platelet function; however, secondary hemostasis may remain unaltered [11]. Given the normal platelet life span of 7-10 days and the absence of alcohol use (19 days) during the hospital course in our case, we cannot assume the role of alcohol intoxication in the rebled status. Similarly, Chen et al [8] reported 2 cases of delayed SDH who, despite undergoing decompressive craniotomy, died postoperatively. On retrospective chart review, his coagulation parameters (PT, INR, and PTT) were within normal limits; however, due to the urgency of the situation, a viscoelastic test (TEG/ROTEM) could not be performed.

Matsuda W et al. [6] described a young, previously healthy male who initially presented with headache 4 days after a minor head injury during a football game, with normal neurological examination and CT imaging. On the sixth day postinjury, he developed suddenly. headache and CT revealed an acute subdural hematoma, which was successfully treated by craniotomy and evacuation, resulting in full recovery.

## Conclusion

Although rare, delayed intracranial hemorrhage in the form of subdural hemorrhage can still happen days or weeks after the initial trauma, without preceding signs or symptoms. Even if the case is diagnosed and stabilized on an emergency basis, the sequelae can be devastating, including mortality. The physician must rule out delayed-onset intracranial bleed in a trauma patient, even if the CT and MRI did not show intracranial bleed initially.

## Author's contribution

KA, GS, TS: Conceptualization, Methodology, Data Curation; KA: Visualization, Writing – Original Draft; MA, AE, HA, RP, GS: Writing – Review & Editing, Supervision.

## Ethics statement

Ethical approval was obtained from the institutional review board (MRC-04-24-807) at the Medical Research Center, Hamad Medical Corporation.

## Patient consent

Written informed consent to publish this case was obtained from the patient representative authorized by the patient and available upon editor request.
